# Supply Chain Challenges in Pharmaceutical Manufacturing Companies: Using Qualitative System Dynamics Methodology

**DOI:** 10.22037/ijpr.2019.2389

**Published:** 2019

**Authors:** Asiye Moosivand, Ali Rajabzadeh Ghatari, Hamid Reza Rasekh

**Affiliations:** a *Department of Pharmacoeconomics and Pharmaceutical Management, School of Pharmacy, Shahid Beheshti University of Medical Sciences, Tehran, Iran. *; b *Department of Industrial Management,* *School of Management and Economics, Tarbiat Modares University, Tehran, Iran.*

**Keywords:** Pharmaceutical supply chain, System dynamics, Performance, SCOR model, Iran

## Abstract

In today’s competitive market environment, pharmaceutical companies have learned that improving supply chain performance is critical to maintain competitive advantages. Forecasting, planning, procurement, financing, stock levels, and marketing strategies are some of the areas in which managers have to decide about them and balance their enter-related effects simultaneously, to achieve organizational goals. This study is based on the results of literature review, experts’ opinion acquisition, and qualitative system dynamics modeling. So, according to method, triangular researches have been considered. The purpose of this research is to explore pharmaceutical supply chain (PSC) challenges and the dynamics behavior of variables playing a special role in PSC. Also, it provides different policies to overcome the challenges. For the first step to reach this goal, several semi-structured interviews with expert supply chain managers are conducted to explore the main challenges. Inaccuracy in forecasting, long lead times, lack of optimum target inventory, and high SC costs are the most important PSC problems. Then, qualitative system dynamics methodology is used to demonstrate the inter-relationship between variables that have impact on challenges. Finally, three strategic policies are recommended including: Collaborative relationship with suppliers, Investment in new technologies, and Information technology (IT) establishment. Consequently, the results can give PSC managers a comprehensive view for decision making and bringing their attention to the importance of feedback behavior of variables in long term and their effects on organizational decisions and goals.

## Introduction

Recently, firms have been increasingly interested in efficient supply chain management (SCM), because they have met extreme competition. This is because of decreasing product life cycle time, varying customer demands, as well as increasing cost of manufacturing and shipment (1). This condition has led many companies to recognize the critical role of SCM to reach organizational goals via speeding up innovations and product launching to the dynamic market, improving customer value, optimizing utilization of resources, decreasing different types of costs such as production, inventory, transportation, and so on, and increasing profitability (2). A supply chain as a dynamic process is usually described as a forward flow of materials and a backward flow of information and funds among multiple operating units both within and between chain members (3). Because of rapid growth rate of technology development, basic supply chain structure has been changed to supply chain network with more complex structure including a higher level of interdependence and collaboration between more entities. Supply chain networks can be used to highlight interactions between organizations; they can also be used to show the flow of information and materials across organizations. Supply chain networks are designed with five key areas: inbound logistics (suppliers), internal logistics (production), outbound logistics (distributors), demand sectors, and shipment assets (4). 

In generic pharmaceutical industry, typical supply chains consist of the following components: manufacturing raw material, manufacturing pharmaceuticals, distributing centers, retail pharmacies/hospitals, and patients (5). Because of the economic changes, pharmaceutical industry member companies have been trying to restructure their supply chains. The pharmaceutical business is a multipart enterprise accompanied by conflicting purposes and several troublesome limitations. A highly regulated setting combined with the life changing nature of the products describe the pharmaceutical industry as a special challenging system (6). As Wang said: “the crucial aim of SCM in pharma industry is to make the right product, for the right customer, in the right amount, at the right time” (7). 

Complicated activities in industrial processes are because of existing a multitude of variables and their non-linear dynamics. Also, developing a systematic model to define such these processes behavior is generally difficult or unfeasible (8). Several different methods have been suggested for modeling supply chains. Most of them are steady-state models based on constant conditions; however, static models are not enough for dynamic specifications of the supply chain due to oscillations, lead time delays, sales forecasting, etc. Owing to this reality, system dynamics (SD) method could be a proper technique for displaying interactions between several factors used in modeling tool.

In this study a wide spectrum of pharmaceutical supply chain (PSC) challenges are discussed and tried to determine the internal PSC problems and the interrelationship between variables which affect PSC performance. The supply chain operations reference (SCOR) model criteria was used for classifying challenges and complicated relationships among various variables are shown using qualitative SD modeling. 

*Literature Review*

*Pharmaceutical supply chain challenges*

With the increasing changes in business environment, firms have to supply high quality products, deliver fast responses, and make their dynamic competencies better. Particularly, the pharmaceutical industry is facing the same challenges that many other industries have experienced in the past. Only the firms that are eager to accept changes and improve their strategies will achieve long term success (9). 

The challenges that pharma companies are involved in are complicated and have an extensive range including political, economic, social, technical, and legal considerations. The pharmaceutical industry is characterized as a group of organizations, processes and actions, engaged in the invention, and innovation of pharmaceuticals; furthermore PSC is made up of corporations to supply and deliver medications which have an important effect on customer satisfaction (10).

Today, efficiency of R and D processes, products’ declining life cycle and patent life exclusivity, increasing generic competition, production compliance, and costs, are some of the major complications that pharmaceutical companies encounter with them (9, 11). A study by Oliver Eitelwein shows that many pharmaceutical companies have to enhance the main supply chain sections including customer satisfaction, forecasting accuracy, inventory level, and total supply chain costs. Also, he states that the complex nature of products and processes are other important matters which can emerge from various number of causes: the large finished good portfolio, wide variety of materials needed, distribution networks, high investment cost and time of developing new products, capacity constraints, and regulatory restrictions (12). 

*Supply Chain challenges related SCOR Perspectives*

Like other industries, the supply chain in pharmaceutical industry initiates with the sourcing of material for production. Active pharmaceutical ingredient (API) along with other inactive materials are planned to formulate in to the standard dosage forms and filled into primary and secondary packages with different configurations. Finished products are transferred from manufactures’ warehouses to distributors, retail/hospital pharmacies, and finally to consumers. In contrast, the flow of data and funds starts from end-users to producers through several channels (13). As mentioned earlier, there are a lot of factors that can affect pharmaceutical industry performance. Discussing all supply chain related variables is not the purpose of this study. Thus, in this research, the Supply Chain Operations Reference (SCOR) is used as a conceptual model to focus on the crucial variables. SCOR model proposed by the Supply Chain Council in makes a helpful structure for performance evaluation and offers standardized definitions for measures and metrics for all members in the supply chain in various industries (2, 14) . Many analytical models in business and engineering fields, have been suggested to handle supply chain operational and design matters (15). While, there are a few holistic models for strategic decisions. Based on Huan survey, “the most promising model for supply chain strategic decision making is the SCOR model developed by the supply chain council” (16). For the assessment and improvement of supply chain management and performance, the SCOR can creates a cross industry structure (17). In the SCOR model, the function of SCM from operational perspective is considered. Recently, several studies have looked over SCOR model and have tried to measure the impact of this model on organization performance (16, 18-20). In the study was conducted by Zhue, the relationship between supply chain process in the SCOR model were proved (21). 

Therefore, this comprehensive model verified by many academics and experts in both academic and business area, was chosen as a frame work in this research to study supply chain problems in pharmaceutical manufacturing companies.

In the SCOR model, supply chain activities are a series of connected inter-organizational processes containing five echelons: plan, source, make, deliver, and return. Each supply chain echelon has individual intra-organizational processes evaluated with five strategic attributes including "supply chain reliability, responsiveness, flexibility, costs, and assets". The first three attributes are related to customer orientation measures (effectiveness) for example delivery performance, while the other two are internal efficiency measures of a company like cash-to-cash cycle time (22).

*Customer orientation perspective*

In this perspective, supply chain capabilities including SC reliability, SC responsiveness, and SC flexibility are considered. Reliability is related to delivery performance when the right product with the right quantity, packaging, and documents is delivered to the right place and customer, at the right time. SC responsiveness is defined as how fast the supply chain can respond to customer demand. The flexibility of SC is the ability to effectively increase or decrease aggregate production or switch rapidly from one product to another in response to customer demand changes to achieve or sustain competitive advantage. This ability can reduce the risk of products destocking arising from unexpected increase demands. Additionally, will render companies needless of stocking up on large quantities of inventory (23).

Nowadays, the common strategy for maintaining competitive advantages is the time-based competition strategy. Supply chain must compress the time required to propose, develop, manufacture, market and deliver its products to provide a respond to customer demands in as short as possible delivery times. Indeed, responsiveness to the market demand is a prerequisite of reliability. Responsiveness can be defined as the ability of the supply chain to respond purposefully and within an appropriate timeframe to customer requests or changes in the marketplace which is also referred to as *agility* (24). Agility paradigm can be noteworthy in the pharmaceutical industry because of many reasons such as reducing the product life cycle, increasing merge and acquisitions, changing customer behaviors, and competitive actions which enforce companies to respond faster (12).

**Table 1:** The SCOR performance attributes with the associated level 1 metrics

**SC Performance**	**SCOR Perspectives**	**Metrics**
Supply Chain Capabilities	Reliability	Delivery performance Fill rate Perfect order fulfillment
	Responsiveness	Order fulfillment lead time
	Flexibility	SC response time Production flexibility
Supply Chain Efficiency	Cost	Total SC cost Cost of goods sold Value added productivity Return processing costs
	Asset Management	Cash to cash cycle time Inventory day of supply Asset turns

**Table 2:** Supply Chain challenges in pharmaceutical manufacturing company

**SC Performance**	**SCOR Perspectives**	**Problems related this metrics**
Supply Chain Capabilities	Reliability	High backorders value (Sales loss) Destocking episodes and inventory oscillation Forecast inaccuracy Delay in supply orders
	Responsiveness	High average manufacturing cycle time
	Flexibility	Lack of on time delivery percentage Lack of response to unpredictable changes Time consuming R and D process
Supply Chain Efficiency	Cost	High inventory cost
	Asset Management	Long cash to cash cycle time Inactive inventory percentage

**Figure 1:**
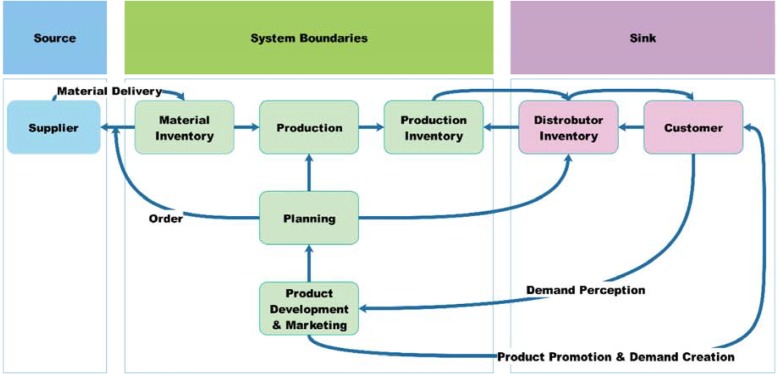
General model boundaries for market oriented pharmaceutical supply chain

**Figure 2:**
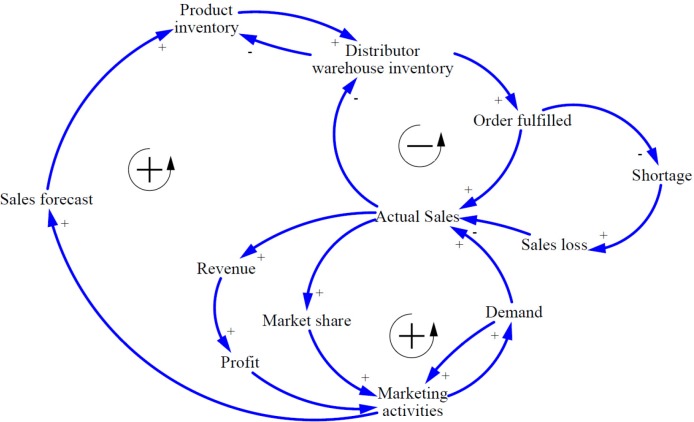
Basic causal loops model

**Figure 3:**
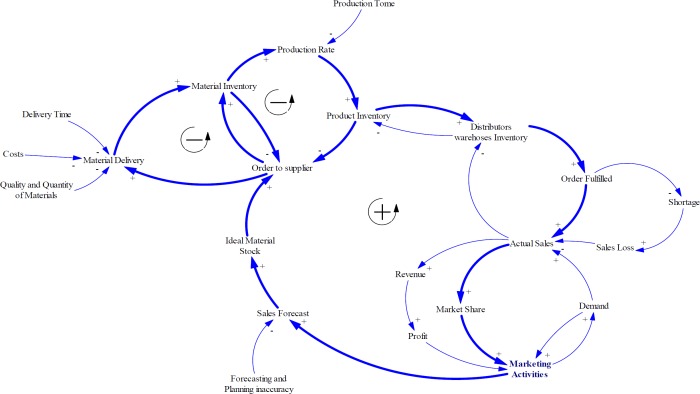
Internal processes: Planning, Sourcing, and Making

**Figure 4:**
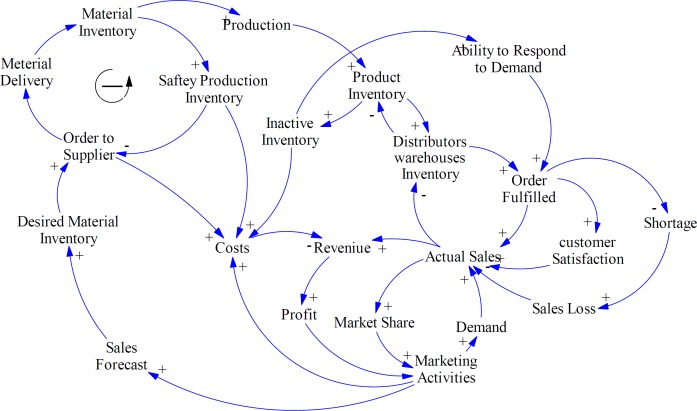
Inventories balancing loops and costs

**Figure 5:**
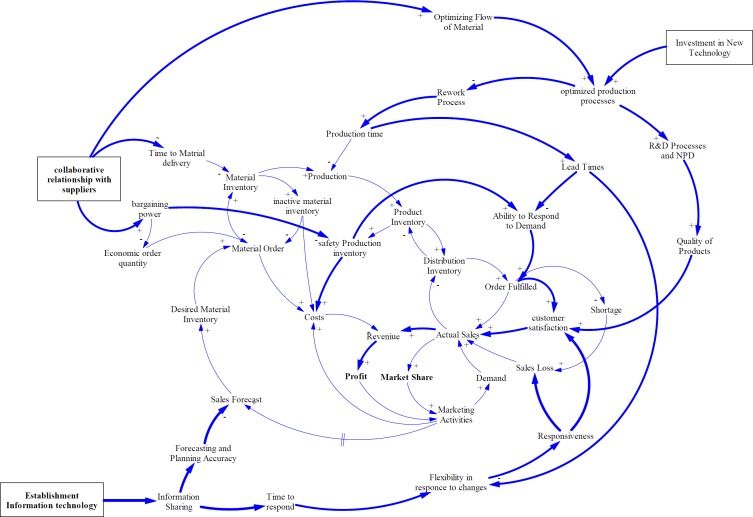
Improving policies for overcoming challenges

*Internal perspective*

Supply chain efficiency is important in this perspective in terms of cost and asset management. With efficient SC management, cost of production as well as inventory and transportation are reduced, and customer service levels are enhanced. All costs related to operating the supply chain such as cost of processes to plan, source, make, deliver, and return, cost of goods sold, direct costs (labor, materials), and indirect costs (overhead) are considered to reach productivity. Cost of operation greatly affects profitability thereby affecting the whole firm performance; hence, it is one of the most important indicators to evaluate efficiency. The more the companies optimize the costs, the higher efficiency they gain (25). Cost reduction is a way in which excellent companies try to create more efficient relationship with partners and other firms to reduced cost of their products**, **reduce internal lead times and work in process inventories, increase forecast accuracy and repeatability, and adopt just in time delivery strategies for their high cost raw materials. By doing these activities indirect costs have been significantly decreasing (26). 

Supply chain assets management is evaluated by three important variables, cash to cash cycle time, return on SC fixed assets, and return on working capital. Cash to cash cycle time is the time period between the point at which a company pays for purchasing material and gains incomes from the products sale in cash. This factor is used to calculate the financing requirements for current and future operations (14). 

## Experimental

*System Dynamics methodology*

Supply chain systems have a dynamic characteristic because of uncertainties in demand, supply, and various logistics methods. Recently, with increasing complexity of supply chain dynamics using system dynamics modeling has become popular (27). As SD models are based on feedback models, causal loop diagrams (CLD) were provided to explain intrinsic feedback of activities. “The feedback loop when a change in something ultimately comes back to cause a further change in the same thing” (28).

*Qualitative system dynamics based on causal loops*

Many authors suggested that CLDs could be used as a conceptual model and help to structure and solve managerial problems (29-31). With the progression of this concept, two parallel methods were formed. The first concept which is termed "*qualitative system dynamics*" was intended to apply all phases of system dynamics besides quantification modeling. The idea of setting up model boundaries has specified responsibilities of managers to prospect how processes might be interacted. Furthermore, it could be combined in to most simulation packages (32). The second concept of CLDs was made as an important part of organizational learning which is named "system thinking". Also, this idea has made managerial perception about the system behavior by concluding, rather than calculating (28). Visualizing dynamic problems with CLDs is an ideal way to bring the decision points and performance measures to the managers’ attention (33).

*System Dynamics modeling processes*

The standard system dynamics method based on Sterman definition is the sequence of activities to study a particular problem (30). These steps are problem definition, dynamic hypothesis, formulation, testing, policy formulation and evaluation. The SD method is used here to take into consideration all variables in terms of both systemic approaches to the variables interactions and flows, and managerial perspective. 

This study tries to explore the pharmaceutical manufacturing supply chain challenges in Iran and find out the relationship between variables affecting these problems by the first two steps of SD method, problem definition, and causal loop diagram. The model provided below is a qualitative model based on extensive literature review and semi-structured interview with experts on pharmaceutical supply chain.

Based on the findings, there are several serious challenges in Iran pharmaceutical industry which have an influence on the competitiveness, profitability, and overall supply chain performance in both external and internal organizations environment. Macro environment troubles include macroeconomics and political situation like sanctions, exchange rate fluctuation, financing issues, lake of proper infrastructure, counterfeit drugs, and lake of transparency and unpredictability of shifts in government economic policies, and highly regulated nature of this business. On the other hand, lack of integration and collaboration between different echelons of supply chain, lack of information visibility, transparency and sharing, high rate of obsolescence machinery, costly and time consuming R and D processes, restricted pricing policy affecting the competitiveness and quality of products, uncertainty in supplying qualified raw materials in their respective required quantity and at the right time lack of effective supplier relationship management are just a number of inter organization problems. 

*Problem definition (Boundary Issues)*

The extent of the model must be defined in any kind of modeling. Conscious decision must be made to clarify where the boundaries of the model are. Otherwise, developing a system model could be ultimately endless. 

Therefore, the first part of the research was undertaken by Semi-structured face to face interviews with 25 expert managers from different pharmaceutical manufacturing companies who have at least 5 year responsibilities in any parts of the supply chain, including marketing, planning, commercial, manufacturing, sales, and financing for clarifying pharmaceutical supply chain structure and model boundaries, as well as their challenges related to SCM.

To address these goals, the interview questions were developed and guided by the wide-ranging literature review and the five main SCOR performance attributes and their associated level 1 metrics (34, 35) Table 1 represents the five performance attributes used within the SCOR model and the associated thirteen level one metrics. 

The interviewees were asked to explain the supply chain structure and processes in their companies and specify the challenges which can cause difficulty in achieving supply chain goals, considering SCOR performance attributes. In addition, the interviewees were given the opportunity to add any other challenges that they believed were relevant for their particular operation. The interviews were recorded digitally and the notes were taken during the interviews. 

The model boundaries and variables included in each sector of the CLDs were based on the notes taken and the audio recordings of interviews. Figure 1 shows the market oriented pharmaceutical supply chain and model boundaries.

In brief explanation, in this industry, the SC Processes initiate by product development and marketing activities as a strategic function. This means that the sales and marketing unit try to find the market needs and change them to demands. They achieve their goals by communicating with physicians authorized to prescribe pharmaceutical products to end users. On the other hand, sales and marketing unit interacts with distribution companies and pharmacies to sell their products. In this way, they can monitor the market as downstream and forecast future demand. Marketing department provides forecast information for planning department to schedule all internal processes including purchasing materials, formulation and making finished products, and distribution in response to market demand. The branded-generic pharmaceutical manufacturing companies also have upstream companies which may have local or international activities, to supply active pharmaceutical ingredients and other inactive materials. Therefore, the supplier selection and supplier relationship are critical tasks for manufacturing companies, they have to evaluated suppliers considering both qualitative and quantitative factors such as quality of materials, delivery performance, their reputation and position in industry, bargaining power, production facilities and capacity, technical capability, geographic location, order fulfillment lead time, net price, total logistics cost etc. (36).

With these descriptions, the model boundaries of this research has determined all challenges related to pharmaceutical manufacturing company processes which can affect them and have ability to improve the processes by changing the policies and decisions. All other concerns related to entities in up and down stream of manufacturer are placed beyond the study boundaries. 

*Dynamic hypothesis*

Based on the interviews, the supply chain challenges of pharmaceutical manufacturing companies are identified and listed in accordance with SCOR model attributes in Table 2.

The basic CLDs of market oriented PSC was developed based on literature review and expert opinions. Figure 2 shows the basic relationship between variables playing a role in the pharmaceutical supply chain.

In general, the most important goal of a *profit-seeking companies *is to maximize stockholder value which can be divided into three sub-objectives including profitability, survival and continuous in existence, and growth. To achieve them, companies must improve financial performance such as - revenue, return on investment, and cash flow - as well as non-financial performance such as - market share, customer satisfaction, number of new products development, and number of employees. 

In market oriented pharmaceutical manufacturing companies, all processes start with product development and marketing activities via market research and analysis to find unmet health needs and create demands through promotional actions to introduce products to the health care professionals. On the other hand, the marketing units can calculate the sales forecasts by observing the market and providing it to planning unit to project the production schedule and finished products inventory in accordance to market demands. The appropriate production inventory can increase the distributors’ warehouses inventory and the orders will be fulfilled completely. At this point, the actual sales can be recorded for manufacturing companies; however, the invoices will be paid with a delay of several months. Increasing actual sales leads to more revenue and profit. On the other hand, market share will be also increased. Ultimately, the marketing departments can benefit from profit to increase marketing promotions to boost demands. There is one subtractive loop to balance the two others reinforcing ones. If the production inventory is not enough to meet the market demands, the distributors could not fulfill orders. By declining order fulfillment rate, shortage and sales loss will occur; as a result, actual sales will decrease. Based on the SCM experts opinions supported by literature, Inaccuracy or anomaly in sales forecast calculating and production capacity limitations can be considered as the causes of inadequate production inventories.

On the other hand, two balancing loops can be added to explain the processes of creating product inventory. As noted earlier, material purchasing plan as well as production plan will be conducted based on sales forecasting. The procurement departments prepare raw materials needed from several supplier companies. There are several factors affecting supply performance of such supplier’s ability to cover orders in terms of quality and quantity of raw materials, cost of raw material and shipment, and delivery time. The material inventory and products inventory have a moderating effect on ordering rate (Figure 3).

As experts asserted in Iran pharma industry, financial and political issues cause some limitations for supplying APIs especially for imported materials. Also, a lack of collaborative relationship strategies with suppliers can create harder condition for pharmaceutical manufacturing companies such as deviation of forecasts, drastic inventory oscillations, and stock-out episodes. Therefore, they buffer a large amount of materials and products inventory. Although, this strategy enhances responsiveness, increases customer satisfaction, and decreases sales loss, it can cause other problems such as the need for more warehouse space and their management, increasing inventory costs, and the risk of material or product expiration (Figure 4).

The other major problem in this business is long lead times including time for new product development, capacity acquisition, procurement, production, distribution, regulatory process, and cash to cash cycle time. The long lead times prevent PSC abilities to be reliable and responsive. This property not only can degrade PSC agility and market share; but, may increase overall costs as well. While, to remain competitive, these firms must reduce costs whereas continuously improve responsiveness. 

After expressing the PSC problems, the dynamic hypotheses are developed. Major challenges which can affect both effectiveness and efficiency of PSC can be briefly summarized as follows: inaccuracy in forecasting, long lead times, lack of optimum target inventory, and high SC costs. 

With such problems, achieving profitability, customer satisfaction, as well as market share would not be possible. There are several improving actions and correcting decisions that have already been experienced in other industries and many researches have showed their effectiveness, and they can be used to overcome these challenges. In the following section, the impact of some of the suggested policies will be discussed on the causal loop model.

Since system dynamics modeling is the interactive process between researchers and participants (manager, owners and associated actors) of problem situation who are experts in their field, after developing the causal loop diagrams the validity of relationship between variables were verified by those pharmaceutical supply chain experts who were participated in the interviews. Then, the suggested policies to mitigate the SC problems were implemented in model and confirmed by them (37, 38). In the next step, the effects of suggested polices were discussed.

## Results and Discussion

*Suggested policies to overcome obstacles*

*Collaborative relationship with suppliers*

Supply chain collaboration has been well defined by Togar as “two or more chain members working together to create a competitive advantage through sharing information, making joint decisions, and sharing benefits which result from greater profitability of satisfying end customer needs than acting alone” (39). With collaboration, the firms can manage the negative impact of “bullwhip effect” and become more responsive to the instability of markets (40). Moreover, the positive impact of collaboration has been proved by several cross sectional studies (41-43). 

When improvement of firm performance is targeted through more effective management of the supply chain, the suppliers’ role becomes important to coordinate supply and demand properly (44). Several researches have been conducted to assess the supplier and manufacturing company relationship and its effects on manufacturing performance. The topics include: supplier selection, supplier alliances and strategic supplier alliances, as well as-supplier management orientation (45-50). 

Long term relationship between manufacturer and its supplier is termed strategic supplier relationship. It is used to help firms reach major benefits. Also, it makes companies more capable of working with a limited number of important suppliers efficiently. Strategically aligning firms will save time and effort (51-54). On the other hand, supply chain collaboration with strategic suppliers empower firms to be more responsive to market fluctuations as a result enhanced capability and the high level of knowledge sharing and communication (55, 56). Strategic partnership helps pull production companies to be more flexible and utilizes lean manufacturing methods needed for pull production by reducing the cycle times (57).

As Figure 5 shows, in this study, the effect of collaborative relationship with suppliers on supply lead time, bargaining power and order quantity has been evaluated. Strategic partnership or trust reduces lead times, safety stocks, economic order quantity, and costs; while, increases ability to respond to demand, customer satisfaction, market share, and profitability. These results are supported by several former studies in other fields. Firm performance is positively linked with activities, in which suppliers are involved in organization operations (58). Also, in different studies the effect of collaboration on resource management, information accuracy, and cost reduction have been proved (59, 60). In addition, assessment and prioritizing of suppliers is the main factor for agility of SC in pharmaceutical companies based on result of the study conducted by Rajabzadeh *et al.* (61).

*Investment in new technologies*

The other improving policy is applying new technology to enhance R and D processes and quality of products, optimize current production processes, decrease rework and wasted material. In one study the author asserted that production technologies can notably improve competitiveness (62). Furthermore, other studies have shown flexibility enhancement by using innovative manufacturing technologies (63, 64). Then, as flexibility is a necessary attribute of pull production businesses, advanced manufacturing technology can improve efficiency and effectiveness of pull production. 

Also, product innovation especially in complex products with unpredictable demand can be increased by new manufacturing technology (65). 

With investment in new and advanced technologies in pharmaceutical companies, flexibility, quality of products, and customer satisfaction are increased. Moreover, by reducing production lead time, responsiveness can be improved. All these behaviors lead to increase actual sale as well as market share. Researches have stated that the environmental uncertainties relate with need for faster adoption new technology to be more flexible and responsive. Logistics executives rank technology as the most important factor in improving SC capabilities (66). 

*Information technology (IT) establishment*

Information is varying between upstream and downstream of supply chain companies. Every entities in SC have to calculate their possible future market demands based on inadequate information obtained from other SC components. Therefore, all partners keep higher amount of inventory to reduce risk of stock-out and preserve responsiveness to market changes. Thus, the costs would be increased (67). Establishment of information technology to make visibility or information sharing among supply chain entities, is one of the critical elements for having an effective SC (68, 69). By using IT, sales forecasting can be accurate, so need for safety or inactive inventory as well as inventory costs will be decreased. Visibility causes less bullwhip effects and increases reliability (70). 

On the other hand, collaborative behaviors have many advantages for all SC partners such as a clear understanding of future demands and realistic plans to respond to demand (71). Researchers have recommended the IT system establishment for information sharing and in this way, enabling collaboration in the supply chain (72, 73). According to the result of Mehralian study, IT is one of the most important factors that can affect coordination in supply chain (58). In addition, using IT for improving information sharing, has positive impact on SC performance (74). 

## Conclusion

To obtaining value for consumers and supply chain network which is the final purpose of supply chain management, supply chain entities must integrate inter and intra organizational processes. A firm’s competitiveness is extremely related to integrated management. Process integration refers to coordination, and resources and information sharing to manage the process cooperatively (70). Chopra and Mendhl enumerated the benefits of SC integration including safety stock and costs reduction, flexibility, responsiveness and quality enhancement, optimum resource utilization (15). However, process integration may have very difficulties in terms of organizational culture, infrastructure and facilities, willing to learn and prepare for changes. Therefore, this model could be a basis for configuration of supply chain system.

Consequently, the policies recommended in this study lead the pharmaceutical supply chain to be more integrated. The collaborative planning, forecasting and replenishment (CPFR) is suggested to implement in pharmaceutical supply chain management to improve replenishment, reduce inventory and backorder value, and decrease procurement and delivery time. 

*Limitation and management implications*

For the first time, the most important internal supply chain challenges in the pharmaceutical market-oriented manufacturing companies were studied in this research. The research ﬁndings in this paper focuses on how a qualitative simulation method can effectively assist decision makers involved in the process of creation policies. The proposed causal loop diagrams can visualize the effects of the activities and decisions on organizational goals; hence, it can help managers to make better decisions and overcome problems. 

In addition, CLDs can be used in other system dynamics modeling as a conceptual frame work to develop stock and flow diagrams and calculate the effects of organizational policies on supply chain challenges quantitatively. Indeed, having such these frame works help organizations to make and record data based on the CLDs variables for more detailed quantitative analysis. However, in this research, interviewees were SC managers in manufacturing companies. For future studies all the participants along the supply chain, including upstream suppliers and downstream customers can be involved. The study is limited to the pharmaceutical industry, and this could limit generalizability of results to other industry types.
